# Subjective cognitive complaints and permanent work disability: a prospective cohort study

**DOI:** 10.1007/s00420-020-01643-1

**Published:** 2021-01-19

**Authors:** Minna Pihlajamäki, Heikki Arola, Heini Ahveninen, Jyrki Ollikainen, Mikko Korhonen, Tapio Nummi, Jukka Uitti, Simo Taimela

**Affiliations:** 1 Faculty of Medicine and Health Technology, Tampere University, Kalevantie 4, Europe, 33014 Tampere, Finland; 2 Terveystalo, Jaakonkatu 3b, 00100 Helsinki, Finland; 3 Faculty of Information Technology and Communication Sciences, Tampere University, Tampere, Finland; 4 Clinic of Occupational Medicine, Tampere University Hospital, Tampere, Finland; 5 Finnish Institute of Occupational Health, Tampere, Finland; 6 Clinicum, Department of Orthopedics and Traumatology, University of Helsinki, Helsinki, Finland; 7 Evalua International, PO Box 35, N02661 Espoo, Finland

**Keywords:** Occupational health care, Self-reported data, Disability retirement, Register data, Subjective cognitive complaints, Fine-Gray model, Cumulative incidence function

## Abstract

**Purpose:**

Work disability (WD) is a medico-legal concept that refers to disability benefits (DB) granted due to diseases. We assessed whether subjective cognitive complaints (SCC)—presenting as self-rated difficulties of concentration, memory, clear thinking, and decision making—predict permanent WD in knowledge-intensive occupations.

**Methods:**

In this prospective cohort study with up to 7-year follow-up, we combined the SCC questionnaire results with reliable registry data on the DBs of 7161 professional/managerial employees (46% females). We excluded employees who were on long-term sickness absence (SA) or had received a DB at baseline. The exposure variable was the presence of SCC. Age and SA before the questionnaire as a proxy measure of general health were treated as confounders and the analyses were conducted by gender. The outcome variable was a granted DB. The cumulative incidence function illustrates the difference between SCC categories, and the Fine-Gray model estimates the predictors of WD during the 8-year follow-up.

**Results:**

The annual incidence of DB was 0.15% in the entire cohort: 0.18% among the females, and 0.12% among the males (*p* = 0.795). The most common primary reasons for permanent WD were mental (36%) and musculoskeletal (20%) disorders. SCC predicted DB in both genders when controlling for age and prior SA. Hazard ratios were 2.9 with a 95% confidence interval 1.4–6.0 for the females and 3.7 (1.8–7.9) for the males.

**Conclusion:**

Subjective cognitive complaints predict permanent WD in knowledge-intensive occupations. This finding has implications for supporting work ability and preventing work disability among employees with cognitively demanding tasks.

## Introduction

Permanent work disability (WD) is one of the greatest societal challenges for the Organisation for Economic Co-operation and Development (OECD) countries (OECD 2010). WD is a medico-legal concept (De Boer et al. 2008), which in Finland is defined as having been granted a work disability benefit (DB). In Finland, 144,600 individuals among the working-age population of 2.5 million (5.7%) have retired prematurely due to permanent WD (Official Statistics, Finland 2018), and there were 19,900 new disability benefit (DB) recipients in 2018, of which 31% were granted due to behavioural and mental disorders (Finnish Centre for Pensions 2018a). The benefits programme of the Social Insurance Institution of Finland (Kela) provides coverage for lost income due to a medically certified sickness for up to 1 year (Kela 2016). Thereafter, the DB scheme, which is operated by pension insurance companies, covers lost income for those eligible. Work ability is assessed on the basis of the employee’s remaining ability to earn an income from work that can reasonably be expected on the basis of his/her education, previous work history, age, housing conditions, and other social factors (Finnish Centre for Pensions 2018b). A DB is granted if, based on the attending physician’s statement, the employee’s ability to work is permanently reduced and the expert panel agrees that the decrease in functional capacity and work ability is due to illness or injury (Finnish Centre for Pensions 2018b). Thus, a granted DB serves as a proxy for permanent WD in the present study.

The share of older people in the workforce is constantly growing (Rechel et al. 2013), life expectancies are rising, and the retirement age is increasing (OECD 2018). Rapid changes are taking place in our knowledge-based working-life (Korunka and Kubicek 2017), which may create a scattered psychosocial work environment that overloads cognitive capacity due to multiple communication and information channels, for example. The term “subjective cognitive complaints” (SCC) refers to difficulties with concentration, memory, decision making, and clear thinking (Stenfors et al. 2013a, b). Cognitive complaints hamper the mental executive capacity to prioritize competing tasks, switch between tasks, monitor multiple sources of data, and resist distractions from the task. SCC have been associated with objective cognitive function in studies that have employed comprehensive SCC measures (OECD 2018). SCC are common in the general and working population and often co-occur with other common psychological health problems (Stenfors et al. 2013a). The prevalence of SCC increases with age (Burmester et al. 2016). In the ageing population, the factors contributing to cognitive decline include disease burden, for example, poor sleep quality and depressive symptoms (LaMonica et al. 2019). In the working population, in addition to other functional syndromes, stress-related conditions often entail exhaustion, sleep problems, and depressive symptoms—i.e. factors that reduce WA in phases that are not characterised by clinical illness (Aasvik et al. 2015). A recent systematic review found evidence of physical (cardiovascular diseases and their risk factors), psychological (insomnia and depressive symptoms), and occupational consequences (job satisfaction, absenteeism, new disability pensions, and sickness absences) due to stress in the workplace (Salvagioni et al. 2017). It has also been suggested that SCC as such reduce WA (Aasvik et al. 2015), but compelling evidence is scarce.

In the present study, we used a SCC questionnaire, which showed predictive validity in our previous study on sickness absence (SA) in knowledge-intensive occupations (Pihlajamäki et al. 2020). The SCC questionnaire presents the results as different risk categories based on self-rated problems with difficulties of concentration, memory, clear thinking, and decision making. The predictive ability of the SCC questionnaire on permanent WD has not studied before. We evaluated whether the SCC questionnaire also predicts permanent WD among respondents from various knowledge-intensive, sedentary occupations. We considered the potential confounding effects of gender, age, and prior SA days as a proxy measure of general health. Our underlining hypothesis was that SCC, indicating hampered cognition in demanding tasks, predict DB.

## Methods

The study design was an analysis of prospectively collected register data. We obtained the questionnaire data and the SA data from one occupational health service (OHS) provider’s registers. The DB data were obtained from the Finnish Centre for Pensions (ETK), which combines DBs under different pension act legislation into one file that is linked to the employee’s career, not to a particular employer, and the coverage of the register is practically 100%. We then combined the data registers using a unique identifier, the Finnish social security code. Data privacy was strictly followed.

The Tampere University Research Ethics Board approved the study (ETL code R16074), and it was conducted in accordance with the Declaration of Helsinki.

The study setting is preventive OHS in Finland within the context of Finnish DB legislation.

## Participants

**Fig. 1:**
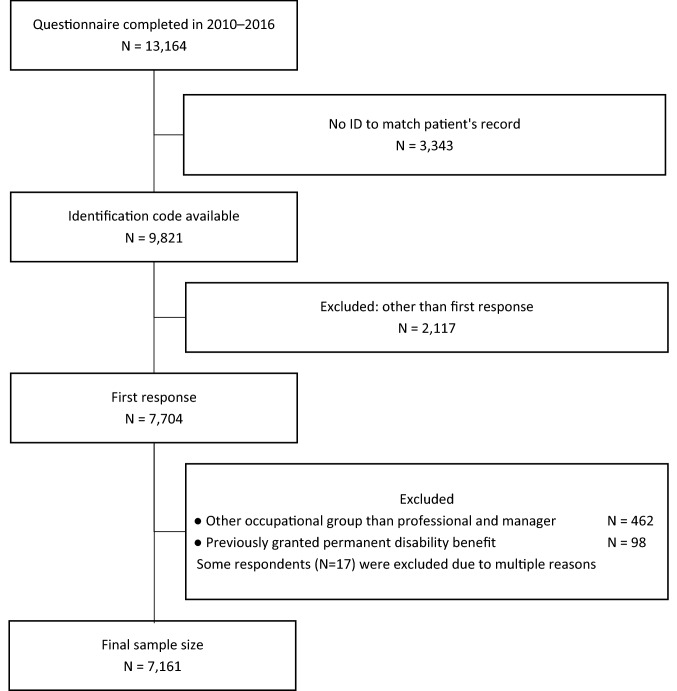
Study flow

The study participants were professional/managerial employees aged 18–68 who had completed the SCC questionnaire (*N* = 13,125) during 2010–2016 as a part of nationwide OHS service provision at one service provider. The information on the archival data of DB from the ETK covered the years 2009–2017. We had access to the complete information on all DB events including the participants’ primary and secondary diagnoses based on the International Classification of Diseases, 10th Revision. The study flow with the participants’ inclusion and exclusion criteria are displayed in Fig. 1.

The questionnaire was completed as a part of an occupational health surveillance programme. Usually, occupational surveillance is conducted every 3 years, and some of the employees had participated in multiple rounds of surveillance during the 8-year follow-up. The exclusion criteria were an unknown identification code (*N* = 3343), other than first response (*N* = 2117), occupational group other than professional/manager (*N* = 462), and previously granted permanent DB (*N* = 98).

The final sample size was *N* = 7161. The exact response rate is not available, because the exact number of invitations was not available in the registry data. In a previous cohort study with the same instrument, the response rate was 64% (Ahveninen et al. 2019).

## Questionnaire

The online questionnaire is used in Finland as a part of preventive OHS by one nationwide provider to recognize employees at risk of exhaustion and to target interventions for those in need. The questionnaire includes a set of nine screening questions derived from various theoretical frameworks to assess psychosocial workload and individual resources for coping. If any of the trigger questions indicates potential psychosocial problems at work, seven additional questions concerning cognitive function are asked. Table 1 shows the topics and cut-off limits of the trigger questions.

**Table 1:** Topics and cut-off limits for the trigger questions

Topic	Cut-off limits*
1. Duration of working hours per week	≥ 45 h/week
2. Time pressure from workload and feeling of strain within the last 2 months	Continuous perception of pressure and job strain
3. Ability to achieve meaningful outcomes at work, which gives satisfaction	Completely disagree
4. Self-perception of overall resources	With the current working tempo, individual resources remain adequate at the maximum for another 6 months
5. Well-being and energy	The last time when felt well and energized was already over 3 months ago
6. Physical condition	Poor
7. Psychological resources	Feeling overloaded, but able to cope
8. Level of energy after a working day	Three or less on a scale from 1 to 10 (1 = extremely tired, 10 = extremely energetic)
9. Sleep difficulties within the last 3 months	At least three nights per week

*If any of the trigger questions met or exceeded the cut-off, the additional seven questions concerning subjective cognitive complaints (SCC) were asked

Table 2 shows the topics for the seven questions that are used to calculate the SCC score. The response options for the SCC categories are (1) I strongly disagree, (2) I somewhat disagree, (3) I somewhat agree, and (4) I strongly agree with the question. The SCC score is calculated as the average of the seven questions. The cut-off limit for the abnormal SCC score, ≥ 2.4, is based on a preliminary non-published study (*N* = 30) where participants responded to the SCC questions and conducted neuropsychological examinations. Cronbach’s alpha for the SCC score in our data was 0.98.

**Table 2:** The topics for the questions that formed subjective cognitive complaints (SCC) score

1. Memory difficulties
2. Difficulties in planning and organizing own work tasks
3. Forgetting agreed issues and work tasks
4. Difficulties in concentration
5. Delays in recollection
6. Disruptions to thinking
7. Difficulties in recollection

## Measurements

### Work disability

The outcome variable was a granted DB as a proxy measure of permanent WD, and it was operationalized dichotomously as a granted DB: yes/no. The mean follow-up time was 3.0 years (SD 1.8, range from 44 days to 7.2 years, median 2.9 years) from the date of the survey response.

DBs in our study consist of four categories as follows: (1) full and (2) partial disability pension, or (3) full and (4) partial rehabilitation subsidy. A DB is granted if the remaining maximum capacity to work is 40% (2/5), as in the case of a full-time benefit; or 60% (3/5), as in the case of a partial benefit. The duration of the DB can be until further notice or for a temporary period. The common requirement in all categories of DB is the permanent nature of the reduction of work ability, i.e. over 1 year.

### Explanatory variable

We classified the results concerning SCC as follows. First, the respondents who did not indicate any problems with the psychosocial screening questions, and therefore were not asked the SCC questions, were classified as belonging to the reference class. Second, we categorized the SCC score into normal/abnormal, based on the a priori cut-off limit. Thus, the exposure variable consists of three categories: (1) reference (no psychosocial load); (2) some psychosocial load, but normal SCC score; and (3) psychosocial load and abnormal SCC score.

### Potential confounding factors

We identified potential confounders in the study as gender (Messing et al. 2003; Messing and Stellman 2006), age (Osmotherly and Attia 2006), general health (Ferrie et al. 2009; Kivimäki et al. 2003), and socioeconomic status (Bouville et al. 2018). We stratified the analyses by gender and included age (four categories) as a potential confounder. Of the available options, we chose to include accumulated SA days before the questionnaire (continuous variable) as a generic measure of health and well-being. Socioeconomic status was operationalized as occupational group and this potential confounder was controlled by the selection of the respondents, who were employees with professional/managerial status only. Other occupational groups (blue-collar workers and clerical employees) were excluded from the study due to small numbers.

### Statistical methods

Baseline characteristics are presented using descriptive statistics. We compared the demographic characteristics of the participants and non-participants by using the *t*-test and Chi-squared test.

We plotted the cumulative incidence function (CIF) to illustrate the difference in DBs between the normal and abnormal SCC score (Kim 2007). We used the Fine-Gray proportional hazards model to estimate how the SCC categories, age, and prior SA affect the probability of events—i.e. a granted DB—prior to a follow-up (Fine and Gray 1999). The Fine-Gray model gives hazard ratio (HR) estimates to describe the relative effect of covariates, which are then associated with the probability of a DB occurring over time. Model 1 is the unadjusted (crude) model and includes only SCC categories (reference/normal/abnormal), and Model 2 is the adjusted model for age and earlier SA days. The estimation of the model parameters was performed using R library cmprsk, with R 3.4.4 software version.

## Results

The average age of the participants was 46.8 (SD 9.8; range 19.2–67.7). Of them, 45% (*N* = 3255) were female. The respondents worked mainly in information and communication technology (48%, of which 62% were males); professional, scientific, and technical activities (23%, of which 60% were males); and public administration, defence, and compulsory social security (9%, of which 29% were males). The corresponding figures in Finland are 4%, 11%, and 7% according to Statistics Finland (Findicator 2018). The excluded respondents were slightly older than average (51.6 years, SD 8.98; *t* 12.3, *p* < 0.005) and a larger proportion of them were men (67.2%; Chi-squared 102.0, *p* < 0.005) compared to the participants.

A total of 85 participants in the cohort were granted a DB an average of 3 years after filling out the questionnaire (range from 44 days to 7.2 years). The overall annual DB incidence was 0.15%: 0.18% among the females and 0.12% among the males (*p* = 0.795). In the Fine-Gray model, which included gender as the exposure variable and age and SA days before the questionnaire, the HR for gender was 1.26 (0.81–1.94; males as the reference).

**Fig. 2:**
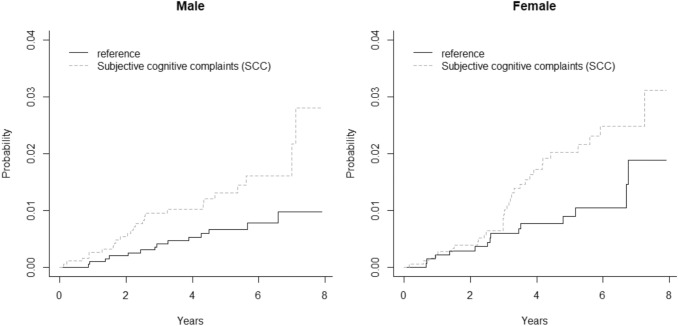
Cumulative incidence of disability benefits over the 8-year follow-up period by subjective cognitive complaints (SCC) among females and males. Estimates are unadjusted crude values with no other explanatory variables. The reference category includes the employees with no psychosocial load and the employees with some psychosocial load, but a normal SCC score

Table 3 shows the causes of DB. Of the participants who received a DB, 31 (37%) had primary diagnoses of a mental disorder and 17 participants (20%) had primary diagnoses of a musculoskeletal disorder. Four participants had both musculoskeletal and mental or behavioural diagnoses simultaneously (5%). Figure 2 presents the cumulative incidence of DB by gender.

**Table 3:** Distribution of causes of disability benefits according to the ICD-10 classification (international classification of diseases, 10th revision)

Cause of disability pension (ICD-10)		First diagnosis		Second diagnosis	
		Total (*N*)	Total (%)	Total (*N*)	Total (%)
**M Diseases of the musculoskeletal system and connective tissue**		17	20	12	14
M40–M54	Spinal disorders	10	12	7	8
	Other musculoskeletal disorders	7	8	5	6
**F Mental and behavioural disorders**		31	36	12	14
F32-F34	Mood (affective disorders)	19	22	5	6
F31	Bipolar affective disorder	3	4	0	0
F25–F29	Schizophrenia, schizotypal and delusional disorders	3	4	0	0
	Other mental and behavioural disorders	6	7	7	8
**C Neoplasms**		12	14	3	4
C50	Malignant neoplasm of the breast	2	2	1	1
C18–C25	Malignant neoplasm of the digestive organs	2	2	0	0
	Other malignant neoplasm	8	9	2	2
**G Diseases of the nervous system**		9	11	5	6
G20	Parkinson disease	3	4	0	0
	Other diseases of the nervous system	6	7	5	6
**I Diseases of the circulatory system**		8	9	4	5
I21–I25	Ischaemic heart disease	2	2	1	1
I60–I69	Cerebrovascular diseases	5	6	0	0
	Other diseases of the circulatory system	1	1	3	4
**Others**		8	9	12	14
H00–H59	Diseases of the eye and adnexa	3	4	3	4
H81	Diseases of the ear and mastoid process	1	1	0	0
Miscellaneous (*N* per category < 3)		4	5	9	11
**No information or second reason not registered**				37	44
Total		85	100	85	100

A pension application may include multiple diagnoses, i.e. several ICD-10 classes

Table 4 shows the predictors of DB. In the Fine-Gray model, the unadjusted (Model 1) HR for DB in the abnormal SCC category was 3.3 (1.7–6.6) for the females and 4.1 (1.9–8.5) for the males. In the fully adjusted model (Model 2), the HRs decreased among both genders, being 2.9 (1.4–6.0) for the females and 3.7 (1.8–7.9) for the males. In the fully adjusted model, the HR for SA days before the questionnaire was 1.013 (1.010–1.017) for the females and 1.018 (1.014–1.021) for the males. In the fully adjusted model, the HR for DB by age was the highest in the 50–60-year age group for females, at 3.6 (1.2–10.5).

**Table 4:** Probability of disability benefit by covariates over time

Explanatory variable	Female			Male		
	*N*	HR	95% CI	*N*	HR	95% CI
**Model 1 (unadjusted)**						
SCC: questions did not open	1415	1.0	Ref	2021	1.0	Ref
SCC: normal	1225	1.2	[0.6–2.6]	1372	1.4	[0.6–3.0]
SCC: abnormal	615	3.3	[1.7–6.6]	513	4.1	[1.9–8.5]
**Model 2 (adjusted)**						
SCC: questions did not open	1415	1.0	Ref	2021	1.0	Ref
SCC: normal	1225	1.2	[0.5–2.7]	1372	1.3	[0.6–3.0]
SCC: abnormal	615	2.9	[1.4–6.0]	513	3.7	[1.8–7.9]
Age < 40	834	1.0	Ref	1187	1.0	Ref
Age ≥ 40 and < 50	952	2.8	[0.9–8.3]	1142	0.8	[0.3–2.1]
Age ≥ 50 and < 60	1225	3.6	[1.2–10.5]	1258	1.9	[0.8–4.6]
Age ≥ 60	244	1.8	[0.3–9.7]	319	1.4	[0.4–5.5]
Sick leave days before the questionnaire		1.013	[1.010–1.017]		1.018	[1.014–1.021]

Subdistribution hazard ratios obtained from the Fine-Gray model describe the relative effect of covariates on the subdistribution hazard function. The covariates in this model can also be interpreted as having an effect on the cumulative incidence function or on the probability of events occurring over time

Model 1 includes the questionnaire subjective cognitive complaints (SCC) score only; Model 2 includes SCC, age, and prior sick leave as a covariate

## Discussion

Subjective cognitive complaints (SCC)—self-rated problems in concentration, memory, clear thinking, and decision making—predicted permanent WD in both genders. It was noteworthy that the accumulation of DBs among those with SCC seems to accelerate only from the third year of follow-up onwards. Thus, the presence of SCC seems to act as an early indicator in identifying employees who are at increased risk for work disability in knowledge-intensive occupations. A diagnosis of a mental or behavioural disorder was present at 50 of the 85 granted DBs (59%) as the first or the second diagnosis, i.e., as the primary reason, or a contributing factor for work disability, respectively. Therefore, we assume that SCC may serve as an early warning sign of mental health problems that may develop later on, unless addressed appropriately at an early stage.

In our previous study in the same cohort, an abnormal SCC score predicted SA days in knowledge-intense occupations during 12-month follow-up (Pihlajamäki et al. 2020). Among those with abnormal SCC score, the ratio of the means of SA days was higher than 2.8 as compared to the reference group with the lowest limit of 95% confidence interval 2.2 (Pihlajamäki et al. 2020). The findings of the present study, in which the primary outcome was WD, are line with our earlier study. Most studies concerning SCC have focused on health-related conditions, such as depression (Amiri and Behnezhad 2019) or stress-related symptoms (burnout/exhaustion) (Salvagioni et al. 2017). Obviously, these health conditions are related to permanent WD (Ervasti et al. 2017; Juvani et al. 2016). To the best of our knowledge, there are no previous studies concerning the relationship between SCC and permanent WD as such. Previous studies that have analysed the relationships between SCC and permanent WD have focused either on a particular illness (Kavaliunas et al. 2017) or general ageing (Burmester et al. 2016).

Our results are in line with earlier findings that the accumulation of SA predicts DB (Kivimäki et al. 2004; Salonen et al. 2018), and that the main causes of DB in Finland are diseases of the musculoskeletal system and mental disorders (Ahola et al. 2011; Dorner et al. 2015; Juvani et al. 2016; Kaila-Kangas et al. 2014; Lahelma et al. 2012; Mäntyniemi et al. 2012; Olesen et al. 2012). By age, the risk of DB was the highest in the 50- to 60-year age group in our study population, though it was not statistically significant among the males. This might be due to the “healthy worker survivor effect”, which means that those who become unwell or unfit during their employment tend to leave working life earlier (Osmotherly and Attia 2006).

A key strength of our study is its register-based, prospectively analysed data from different industries among knowledge-intense occupations. We were able to control the key potential confounders, namely age and gender. A previous SCC study also found significant gender interaction (Stenfors et al. 2013a). We performed the analyses separately for males and females due to potential interactions between gender and other covariates, as suggested earlier (Messing and Stellman 2006).

The quality of the archival DB data from the Finnish Centre for Pensions is good in terms of coverage, accuracy, and consistency over time, and no data are lost to follow-up (Finnish centre for pensions 2018b; Kela 2019). We combined all four DB categories as one as the proxy measure for WD: this way no data was lost and virtually all the DB recipients had had at least 1 year of sickness allowance before the granted DB. The follow-up period continued for at least 15 months after the questionnaire had been completed. Sickness allowance is paid for a maximum of 1 year after the onset of WD, and the DB decision is typically made immediately after the sickness allowance period. Thus, the minimum follow-up period of 15 months was long enough to detect all new potential DB recipients.

We chose to use the Fine-Gray model to estimate the effect of the covariates on the rate at which WD occurs. Perhaps the model was not able to deal with all the complexity associated with our data, but among computationally feasible approaches it is more appropriate than Kaplan–Meier survival analysis, for example, as the latter tends to overestimate the cumulative incidence of health-related events (Lacny et al. 2018).It was also easier to add variables to the Fine-Gray model than to Kaplan–Meier. More importantly, we prefer talking about cumulative hazards over “survival at work” conceptually. However, interpretation of the HR estimates from the Fine-Gray model is not straightforward. We recommend interpreting the covariates as having an effect on the incidence of WD (i.e. on the CIF), but the magnitude of the relative effect of the covariate on the subdistribution hazard function is different from the magnitude of the effect of the covariate on the CIF. One can still conclude that if a variable increases the subdistribution hazard function, it will also increase the incidence of the event, but one cannot infer that the exact magnitudes of the two effects are the same (Austin and Fine 2017).

Cronbach’s alpha for the SCC was 0.98 indicating excellent internal consistency. In our previous study, the SCC questionnaire also showed predictive validity for SA (Pihlajamäki et al. 2020). Nevertheless, the lack of proper psychometric validation of the SCC questionnaire may be considered as a limitation of the present study.

Another limitation of the study is the potential selection bias due to differences between respondents and non-respondents. The “healthy worker effect” may be present, since the health of employed people is generally better than that of the unemployed population, and employees with a worse health level may have not responded (Chowdhury et al. 2017). A similar bias would potentially result from a “healthy worker survival effect”, which means that employees with problems with health and well-being are likely to drop out of working life (Nordström et al. 2016). Moreover, we did not include those who had already been granted a DB before the questionnaire. All this might underestimate the associations. On the other hand, it may also be possible that the healthiest employees did not respond to the questionnaire, which would have an opposite effect on our estimates.

The study population consisted solely of professionals/managers in knowledge-intensive sedentary occupations. Cautious generalizations can only be made to other professional groups. People from outside working life were not involved in our study. As we did not analyse statutory accident insurance data, we cannot draw conclusions about the reasons for DB due to accidents at work, occupational diseases, or traffic accidents.

Work ability and disability are complex and multifactorial phenomena determined by personal, socio-demographic, and lifestyle- and health-related factors, as well as organisational determinants, healthcare management, and legislation (Loisel et al. 2001). In most countries with disability pension schemes, permanent WD is usually due to a chronic disease (De Boer et al. 2008) that reduces functional capacity and work ability (OECD 2010). Some DB criteria are comparable between countries and legislation, such as requirements for a health condition in relation to work and the permanence of the condition (De Boer et al. 2008). As the implementation of the legislation varies across countries (OECD 2010), the results of our study must be interpreted with caution in the international context. However, we assume that the phenomenon itself—that subjective cognitive complaints predict WD—manifests in different medico-legal contexts.

The annual incidence of DB was only 0.15% in the entire cohort and the three-fold relative risk is small in terms of absolute risk increase. However, since WD is so costly for society (OECD 2010) and burdensome for the disabled individuals themselves, prevention of permanent WD is important. Moreover, as the working population is ageing, and at the same time working life is becoming increasingly demanding cognitively and psychosocially (Gijselaers et al. 2017; Singh-Manoux et al. 2012), it is important to identify the psychosocial predictors of WD to determine how to prevent ill health and subsequent WD among professional/managerial employees. One of the primary tasks of OHS in Finland includes protection of employees’ work ability, for which purpose early identification of WD risk would be desirable and therefore instruments to tap risks are developed in OHS.

Further research is needed to understand the causal and mediating pathways between psychosocial load, cognitive performance, SCC, illnesses, and permanent WD. The effectiveness of targeting health surveillance among risk groups also need further research.

Our results indicate that subjective cognitive complaints predict permanent work disability among knowledge-intensive occupations in cognitively demanding occupations and therefore seem to act as an early indicator of future work disability risk. This finding has implications for supporting work productivity among employees with cognitively demanding tasks.

## Data Availability

No additional data are available due to data privacy reasons.
